# Prenylated Benzophenones from *Vismia Guianensis* Reduced Nematode Growth and Chemotaxis

**DOI:** 10.2478/jofnem-2022-0054

**Published:** 2023-01-08

**Authors:** Carresse Gerald, Rick-Kia Howard, Rachael Adesina, Seon Hamer, Omar E. Christian

**Affiliations:** 1 Department of Earth, Environmental and Geospatial Science, North Carolina Central University, Durham, NC 27707 North Carolina; 2 Department of Chemistry and Biochemistry, North Carolina Central University, Durham, NC 27707 North Carolina; 3 Department of Environmental Studies, University of Guyana, Turkeyen Campus, Greater Georgetown, Guyana South America

**Keywords:** anthelminthic, behavior, biopesticides, *Caenorhabditis elegans*, interaction, Vismia guianensis

## Abstract

Biopesticides are generally considered a safer and more environmentally friendly alternative to conventional pesticides. Plant metabolites display a range of pest specific activity ranging from antimicrobial to larvicidal and nematocidal. We herein describe the evaluation of a Guyanese collection of *Vismia guianensis* (Clusiaceae) for anthelmintic activity. The bioassay-guided evaluation of the hexane extract yielded the new prenylated benzophenone 8,9-epoxyvismiaphenone F (**1**). The final structures were elucidated based on spectral analysis and comparison to the known metabolite. To evaluate the anthelmintic efficacy of these compounds, *Caenorhabditis elegans* were exposed to the compounds via a ring assay model. Post-exposure, the numbers of live *C. elegans* in the compound (middle), bacteria ring were recorded for 3 d, as well as the total number of live worms for each plate. Compound **1** reduced *C. elegans*’ overall growth and reproduction, suggesting that these prenylated benzophenones may hold some promise as natural pesticides.

Parasitic nematodes can burden livestock and the intense usage of anthelmintic compounds has led to anthelmintic resistance (AR) (Ihler, 2010; Peña-Espinoza et al., 2018; Fissiha and Kinde, 2021). Parasitic helminths not only affect cattle, goats, sheep, and horses but can also burden humans and companion animals (Holden-Dye and Walker, 2014). Health impacts associated with parasitic helminth infections include production losses and death in livestock, weight-loss, anemia, and death in companion animals, and loss of life in humans (Kotze et al., 2020). *Caenorhabditis elegans* is a model organism and nematode and has been compared to other parasitic helminths. Stasiuk et al. (2019) suggest that similar xenobiotic biotransformation occurs in the parasitic roundworm *Haemonchus contortus* and *C. elegans* when exposed to benzimidazoles. *C. elegans* were utilized in this study because of the ease of culture and maintenance in a laboratory setting, their 3-d life cycle, and the extensive studies that have been conducted on this microscopic roundworm.

Biopesticides include plant natural products, like d-limonene isolated from fruit peels and azadirachtin obtained from *Azadirachta indica* (neem), which are currently commercially available and used against a variety of plant pests including root-knot nematodes (National Research Council, 2002; Seiber et al., 2014). Plants in the Clusiaceae family produce a range of biologically active metabolites including terpenes, and prenylated benzophenones (Acuna et al., 2009). The extracts of *Clusia fluminensis* and *Clusia hilariana* inhibited the development of *Dysdercus peruvianus* nymphs (Duprat et al., 2017). However, the activity was confined to the terpenoid fractions, with only modest activity in the fractions harboring the prenylated benzophenones. Clusianone and nemorosone, respectively, were identified from these studies as the predominant benzophenone in these plants. To study biologically significant metabolites from Clusiaceae, we isolated compounds from *Vismia guianensis* (Clusiaceae). We report herein the activity of the new prenylated benzophenone, 8,9-epoxyvismiaphenone F (**1**), on *C. elegans* chemotaxis and reproduction. We also investigated whether the above-mentioned isolated compound impacted acetylcholinesterase activity. This pathway was chosen because many anthelmintic drugs target the nervous system of nematodes and more specifically nicotinic acetylcholine receptors (Sleigh, 2010). Levamisole and pyrantel are anthelmintic drugs commonly used in veterinary medicine. We hypothesized that nematodes exposed to 8,9-epoxyvismiaphenone F (**1**) would reduce chemotaxis and reproduction due to impaired acetylcholinesterase activity.

## Materials and Methods

### General experimental procedure

All 1D and 2D NMR spectra were recorded in CDCl_3_ on a Bruker AVANCE III NMR Spectrometer (Bruker BioSpin, Billerica, MA, USA) at 400 MHz for ^1^H and 100 MHz for ^13^C. LCMS was performed on a reversed-phase analytical column (4.6 mm × 250 mm, 5 μm) using a photodiode array (PDA) detector (Agilent, Santa Clara, CA, USA) and with an electrospray single quadrupole mass spectrometer. High-resolution mass measurements were obtained on an Agilent 6230 ESI_TOF (Agilent, Santa Clara, CA, USA) mass spectrometer. The samples were run in positive mode ionization with a capillary voltage of 4,000 V. Drying gas (nitrogen) temperature was 325°C delivered at 10 L/min and the fragmentation voltage was set to 150 eV. MPLC separation was performed on a Reveleris system (Buchi, New Castle, DE, USA) equipped with a UV and ELSD detectors. All solvents were HPLC grade with 0.1% TFA or ACS grade.

### Collection

The leaves and stems of *Vismia guianensis* were collected along the Soesdyke-Linden Highway, Demerara, Guyana (6° 08´33.33´´ N, 58° 13´17.3´´ W) in April 2018. A voucher specimen (VG18A) is preserved in our laboratory in the Department of Chemistry and Biochemistry at North Carolina Central University.

### Extraction and isolation

The air-dried and pulverized leaves and stems (1.2 kg) were sequentially extracted with hexane (3 × 4 L), EtOAc (3 × 4 L), and MeOH (3 × 4 L). Evaporation of the solvents yielded three crude extracts: hexane (150 g), EtOAc (80 g), and MeOH (60 g). A portion of the hexane extract (50 g) was absorbed unto silica gel and subjected to flash chromatography using an EtOAc-hexane gradient (0%–100%), yielding seven major fractions (A–G). Further purification of fraction E via normal phase chromatography eluting with mixtures of EtOAc-hexane yielded compound **1** (11 mg).

### Synthesis of clusiaphenone B (2)

The synthesis was carried out as detailed previously (Qi and Porco, 2007). To a mixture of 2,4,6-trihydroxybenzophenone (460 mg, 2.0 mmol) and potassium hydroxide (240 mg, 4.3 mmol) in water (1.5 mL), prenyl bromide (485 μL, 4 mmol) was added for over 10 min. The mixture was kept at 0**°**C while stirring for 2 h. A yellow precipitate was formed from the reaction, which was then quenched with HCl. The reaction mixture was extracted with ethyl acetate (3 × 10 mL). The organic layer was washed with brine (2 × 10 mL) and dried over anhydrous sodium sulfate (Na_2_SO_4_). The organic layer was purified using MPLC to yield clusiaphenone B, whose structure was confirmed by referring to the literature (Delle Monache et al., 1991; Qi and Porco, 2007).

### Caenorhabditis elegans inoculation and culture

*Caenorhabditis elegans* (N2 strain), *E.coli* (K-12 strain), and Nematode Growth Agar (NGA) were all purchased from Carolina Biological Supply Company (Burlington, NC). NGA is the medium used to grow and maintain the nematodes. *E.coli* was aseptically inoculated onto NGA plates and incubated overnight at 37**°**C. Post-incubation, nematodes were inoculated onto bacteria lawn plates and maintained at 25**°**C until experimentation time.

### Three-day nematode growth assay

**Figure 1:**
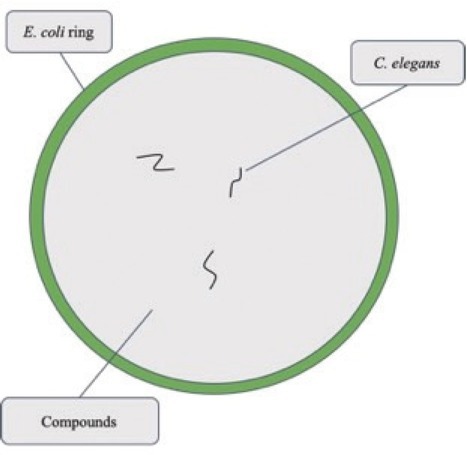
Ring assay plate.

The nematode ring assay was performed as detailed previously (Gerald et al., 2022). Nutrient agar plates were inoculated with *E. coli* in a ring around the periphery of the plate and incubated at 37°C (Fig. 1). Following the incubation of *E. coli*, the plates were inoculated with 30–40 *C. elegans* using ×4 magnification on a dissecting microscope for each ring assay plate. Nematodes were picked from a nematode growth plate (nematodes on a bacterial lawn used growth and maintenance) using a sterile toothpick and placed in the middle of the appropriate ring assay plate. Nematodes were exposed to DMSO (vehicle control), 8,9-epoxyvismiaphenone F (**1**), clusiaphenone B (**2**), or 2,4,6-trihydroxybenzophenone (**3**) in the middle of the ring assay plate. DMSO was utilized as a vehicle control because the compounds (**1**, **2**, and **3**) were reconstituted using DMSO. In the control group, nematodes were not exposed to any compound in the middle of the ring assay plate. The plates were stored at room temperature and live nematodes in the middle and bacterial ring were counted each day for 3 d. The total number of live nematodes were recorded each day for 3 d and is reported as growth.

### Nematode preservation

Approximately 150 nematodes were collected from the ring assay plates following the 3 d of the ring assay and preserved using 100-mM Na_2_B_4_O_7_, 1-mM NaN_3_, and 1-mg/mL BSA, pH 8 and stored at –20**°**C.

### Acetylcholinesterase extraction and assay

Preserved nematodes from previous exposures were thawed on ice. Tris HCL buffer (50 μL) was added to each of the tubes containing nematodes to lyse nematodes. Upon lysing nematodes, tubes were centrifuged at 1,000 RPM for 10 min and supernatants were retrieved and stored at 4°C. The remaining nematode pellets were collected and stored at –20°C. The resulting supernatant was utilized in the acetylcholinesterase assay purchased from Abcam (Waltham, MA, USA). The methods used were according to the manufacturer’s protocol.

### Statistical analysis

The ring assay experiments were conducted in triplicate at independent times for reproducibility assessment. In each of the three experimental replicates, three ring assay plates were used for the control group, which consisted of no compound and DMSO (vehicle control). DMSO was used on another three ring assay plates. Three ring assay plates were used for each of the treatment groups, which consisted of the compounds: 8,9-epoxyvismiaphenone F (**1**), clusiaphenone B (**2**), and 2,4,6-trihydroxybenzophenone (**3**). A total of three ring assay plates were inoculated with nematodes in the following treatment groups for each of the three experimental replicates: control, DMSO, 8,9-epoxyvismiaphenone F (**1**), clusiaphenone B (**2**), and 2,4,6-trihydroxybenzophenone (**3**).

A one-way ANOVA and Tukey’s multiple comparisons post-hoc test were employed via Graphpad Prism version 9 (San Diego, CA, USA) to analyze nematode chemotaxis (nematodes present in the *E. coli* ring, in the middle of the plates, in DMSO or previously mentioned compounds) and growth (total number of live nematodes on the entire plate) (Fig. 1) (Gerald et al., 2022). Data presented for chemotaxis, growth, and AChE include three separate, independent experimental replicates. Each of the experimental replicates contained three ring assay plate replicates, for a total of nine replicates per treatment group (*n* = 9).

**Figure 2:**
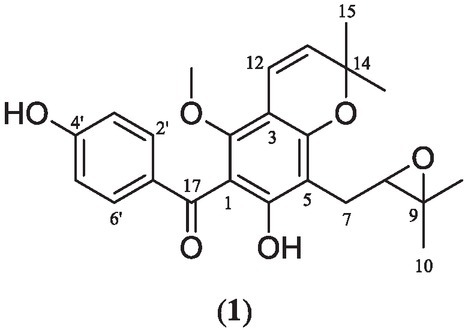
Compound isolated from *Vismia guianensis*. 8,9-epoxyvismiaphenone F (**1**).

### Results and Discussion

The bioassay-guided fractionation of the hexane extract resulted in the isolation of the new derivative 8,9-epoxyvismiaphenone F (**1**). Compound **1** displayed a molecular formula of C_24_H_26_O_6_ by HRESIMS, suggesting 12 degrees of unsaturation, and inspection of the ^13^C NMR showed 22 discrete signals (Table 1). The ^1^HNMR displayed an AA’BB’ system (H-1¢/6¢ 7.70 ppm and H-2¢/4¢ 6.80 ppm, *J* = 8.8 Hz). The ^13^C NMR data also confirmed the presence of a trioxygenated aromatic ring and a benzoyl group consistent with the basic 2,4,6-trihydroxybenzophenone core observed in the vismiaphenone family of compounds (Hussain et al., 2012). The NMR spectra of compound **1** showed several similarities to vismiaphenone F, possessing a para-substituted ring A, a fully substituted ring B, and a 2,2 dimethyl chromene moiety. Analysis of the 2D NMR spectra (HSQC, HMBC, and COSY) revealed that the 2-methyl-2-butenyl group had been oxidized to the epoxide (d_C-8_ 69.0 ppm, d_C-9_ 77.6 ppm), and additionally, significant cross peaks were observed between H-8 (3.70 ppm) and C-5, C-7, and C-11.

**Table 1:** ^1^H and ^13^C NMR data for 8,9-epoxyvismiaphenone F (1) in CDCl_3_.

Position	δ_C_	δ_H_ (J in Hz)
1	107.5	
2	160.3	
3	103.8	
4	153.1	
5	110.4	
6	152.9	
7a	26.0	2.65 (dd, 17.3, 5.6)
7b		2.90 (dd, 17.3, 5.2)
8	69.0	3.70 (dd, 5.2, 5.6)
9	77.6	
10	21.6	1.10 (s)
11	24.2	1.2 (s)
12	116.8	6.50 (d, 9.9)
13	127.4	5.52 (d, 9.9)
14	77.6	
15	28.0	1.44 (s)
16	28.1	1.44 (s)
17	193.9	
1′	131.6	
2′	132.1	6.80 (d, 8.8)
3′	115.2	7.75 (d, 8.8)
4′	160.3	
5′	115.2	7.75 (d, 8.8)
6′	132.1	6.80 (d, 8.8)
OCH_3_	63.4	3.67 (s)

### Anthelmintic activity of compounds 8,9-epoxyvismiaphenone F (1), clusiaphenone (2), and 2,4,6-trihydroxybenzophenone (3)

**Figure 3:**
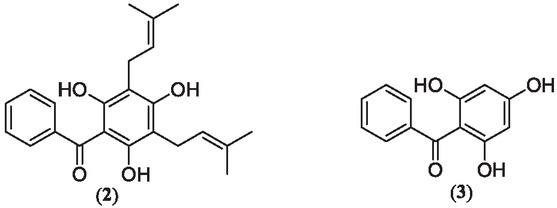
Clusiaphenone B (**2**) and benzophenone (**3**).

To determine if the nematodes’ ability to engage in chemotaxis had been impaired, the nematodes in the bacterial ring were counted on day 3 post-exposure to the compounds **1**, **2**, and **3** (Fig. 3) in the ring assay model. The nematode chemotaxis was measured in response to compounds **1** as well as the metabolites clusiaphenone B (**2**), which we prepared, and commercially available biosynthetic precursor 2,4,6-trihydroxybenzophenone (**3**). The well-studied nematode *C. elegans* was utilized in this study as a model for parasitic nematodes. This is an advantageous model because it has been used in anthelmintic research for over 40 years (Hahnel et al., 2020). It is also preferable over parasitic species because parasitic species need a host to maintain their life-cycle, which can complicate quantitative studies (Holden-Dye and Walker, 2014).

**Figure 4:**
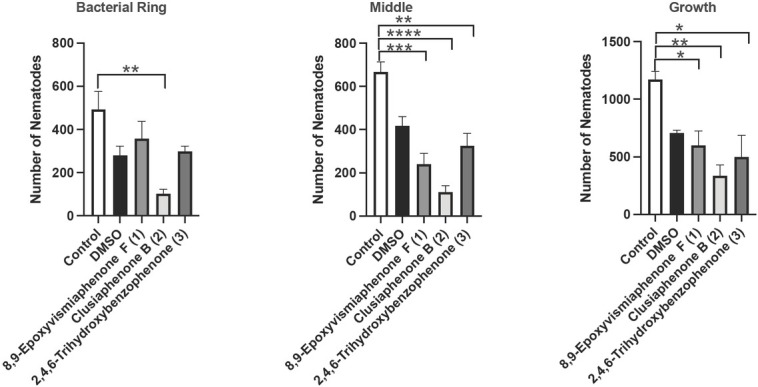
Effects of 8,9-epoxyvismiaphenone F (**1**), clusiaphenone B (**2**), and 2,4,6-trihydroxybenzophenone (**3**) on nematode chemotaxis and growth. **(A)** Nematodes in the bacterial ring. **(B)** Nematodes in the compound. **(C)** Nematode growth on day 3. Chemotaxis and growth decreased with 3-d exposure to 8,9-epoxyvismiaphenone F (**1**), clusiaphenone B (**2**), and 2,4,6-trihydroxybenzophenone (**3**). Nematodes were placed in the middle of nutrient growth agar plates in compounds (**1**, **2**, **3**) for 3 d. Nematodes in the bacterial ring and middle of ring assay plates were counted on day 3. Clusiaphenone B (**3**) decreased the number of nematodes in the bacterial ring compared to control on day 3 when conducting a one-way ANOVA (*P*-value <0.05). 8,9-Expoxyvismiaphenone F (**1**) and 2,4,6-trihydroxybenzophenone (**3**) did not significantly decrease nematodes in the bacterial ring on day 3 when compared to control (*P*-value >0.05). 8,9-Epoxyvismiaphenone F (**1**), clusiaphenone B (**2**), and 2,4,6-trihydroxybenzophenone (**3**) decreased nematodes in the middle of ring assay plate when compared to control on day 3, and the corresponding *P*-values are 0.0005 (***), <0.0001 (****), and 0.0026 (**), respectively. On day 3, all live nematodes on ring assay plates were counted and the number demonstrates growth. 8,9-Epoxyvismiaphenone F (**1**), clusiaphenone B (**2**), and 2,4,6-trihydroxybenzophenone (**3**) decreased nematode growth when compared to control on day 3, and the corresponding *P*-values are 0.0314 (*), 0.0028 (**), and 0.012 (*), respectively. Significance was determined under a one-way ANOVA and Tukey’s post-hoc test. Data are presented as the mean ± SEM for A–C for three independent replicates, each containing three ring assay plates per treatment group, *n* = 9.

When comparing the number of nematodes in the control and vehicle control/DMSO groups for growth and chemotaxis, as expected, no significant differences were found (*P*-values were 0.0885 and 0.1308, respectively), which indicates that there was no effect of DMSO on nematode growth and chemotaxis. No significant difference was found between DMSO and compounds **1**, **2**, and **3** when analyzing growth and chemotaxis. However, the new metabolite **1** decreased nematodes in the middle of the plate when compared to the control, *P*-value = 0.0005 (Fig. 4B), although compound **1** did not inhibit the number of nematodes in the ring on day 3. However, 8,9-epoxyvismiaphenone F (**1**) decreased overall nematode growth when compared to the control, *P*-value = 0.0314 (Fig. 4C). The *C. elegans* life cycle is 3 d, and so observing compound **1** inhibiting nematode growth speaks to the nature of this compound being anthelmintic. Chemotaxis occurs as cells or organisms respond to chemical signals. *C. elegans* occurs naturally in the environment, being present in soil, sediment, and water, where it seeks out bacteria to consume by utilizing chemotaxis. In the case of plant-parasitic nematodes, they use chemotaxis to locate host-plants. Impairment of this is a way to slow the ability of parasitic nematodes to damage vegetation and tissues of host organisms. Clusiaphenone B (**2**) decreased chemotaxis (*P*-value = 0.0043) and growth (nematodes in the ring and middle of ring assay plate) (*P*-value = 0.0028) when compared to the control (Fig. 4A–C). The commercially available biosynthetic precursor of prenylated benzophenone family of metabolites, 2,4,6-trihydroxybenzophenone (**3**), caused a significant decrease of nematodes in the middle of the ring assay plates when compared to the control, *P*-value = 0.0026 (Fig. 4A), but did not affect the number of nematodes in the ring (Fig. 4B). 2,4,6-Trihydrobenzophenone (**3**) also inhibited nematode growth on day 3 when compared to the control, *P*-value = 0.0120 (Fig. 4C), suggesting that the presence of the prenyl groups retards the anthelmintic activity. Nematode growth was inhibited by 8,9-epoxyvismiaphenone (**1**), clusiaphenone B (**2**), and 2,4,6-trihydroxybenzophenone (**3**). The decline in growth in the clusiaphenone B (**2**) treatment group is a result of the inhibition of chemotaxis; if the nematodes are unable to move across the chemical gradient to their food source, then it is even harder to self-reproduce or mate because access to the food source supplying them with energy is no longer available. Nematodes in the ring assay plates treated with 8,9-epoxyvismiaphenone F (**1**) and 2,4,6-trihydroxybenzophenone (**3**) were not significantly different from the control group but had decreased numbers of nematodes in the middle of the ring assay plates, suggesting nematodes’ chemotactic behavior was not impaired but reproduction and growth were slowed.

### Acetylcholinesterase of compound 8,9-epoxyvismiaphenone F (1), clusiaphenone (2), and 2,4,6-trihydroxybenzophenone (3)

**Figure 5:**
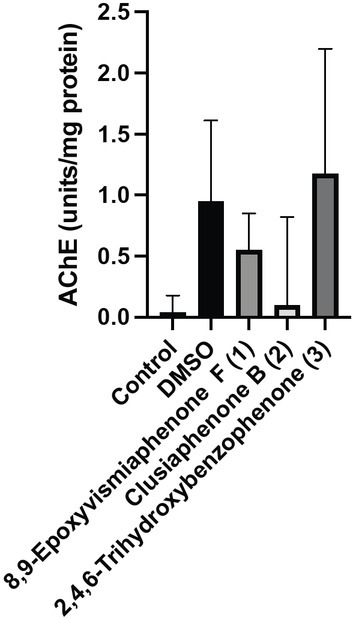
Effects of 8,9-epoxyvismiaphenone F (**1**), clusiaphenone B (**2**), and 2,4,6-trihydroxybenzophenone (**3**) on acetylcholinesterase activity. 8,9-Epoxyvismiaphenone F (**1**), clusiaphenone B (**2**), and 2,4,6-trihydroxybenzophenone (**3**) had no effect on nematode acetylcholinesterase activity. *C. elegans* were exposed to compounds for 3 d. All nematodes were preserved and subjected to acetylcholinesterase activity assay. No significant difference was found when test compounds were compared to the control one-way ANOVA and a Tukey’s post-hoc test (*P* > 0.05). Data are presented as the mean ± SEM for A–C for three independent replicates, each containing three ring assay plates per treatment group, *n* = 9.

While the exact mechanism of action of these compounds is unknown, several prenylated benzophenones including 7-epiclusianone were found to display moderate acetylcholinesterase (AChE) activity (Zheleva-Dimitrova et al., 2013). Due to the important role of AChE inhibitors in current nematicides, it was imperative to analyze the ability of the compounds **1**, **2**, and **3** to modulate expression of acetylcholine, which is a major excitatory neurotransmitter controlling motor activities in the nematodes (Ballivet et al., 1996). Acetylcholine is highly conserved and has central functions in most nematode species (Harder, 2016). Also, the majority of anthelmintic drugs modulate nicotinic acetylcholine receptors (Hahnel et al., 2020). Using the AChE activity assay, it is determined that 8,9-epoxyvismiaphenone F (**1**), clusiaphenone B (**2**), and 2,4,6-trihydroxybenzophenone (**3**) did not inhibit AChE activity (Fig. 5). No significant differences were found between treatment groups. In previous studies, several thousand compounds were screened against parasitic and non-parasitic nematodes, and molecules that killed *C. elegans* were 15 times more likely to kill parasitic nematodes compared to other selected molecules (Reynolds et al., 2011, Burns et al., 2015). This confirms that *C. elegans* are still a good model for anthelminthic resistance despite there being no significant differences between treatment groups when analyzing AChE activity. The broad-spectrum class of anthelminthic imidazothiazoles causes spastic paralysis in *C. elegans* and the parasitic *Ascaris suum* (Holden-Dye et al., 2014). Although no effects were noted pursuant to utilizing the AChE activity assay, acetylcholinesterase could still be affected. In the study conducted by Garcia et al. (2007), [^3^H] benzophenone interacts with the nicotinic acetylcholine receptor. Analyzing the acetylcholine at the genetic and protein levels will allow further elucidation of the mechanism of decreased chemotaxis and growth.

## Conclusion

In summary, 8,9-epoxyvismiaphenone F (**1**) can reduce the growth and chemotaxis of the model nematode, *C. elegans*, but further research is needed to substantiate the validity of the proposed mechanism of action. This compound shows some promise as a biopesticide against parasitic roundworms. In future studies, we will compare the efficacy of these *V. guianensis* prenylated benzophenones with commonly used nematicides such as imidazothiazoles and levamisole, as well as analyze common AChE genes in exposed nematodes. Also, future research will utilize common parasitic nematodes to distinguish genomic differences between the commonly used models of *C. elegans* and parasitic species.
